# Editorial: One Health: The Well-being Impacts of Human-Nature Relationships

**DOI:** 10.3389/fpsyg.2019.01611

**Published:** 2019-08-20

**Authors:** Eric Brymer, Elizabeth Freeman, Miles Richardson

**Affiliations:** ^1^Australian College of Applied Psychology, Sydney, NSW, Australia; ^2^Psychology, Sociology & Politics, Sheffield Hallam University, Sheffield, United Kingdom; ^3^Human Sciences Research Centre, University of Derby, Derby, United Kingdom

**Keywords:** environment, psychological health, psychological well-being, nature, physical activity

This special edition responds to two interrelated issues confronting humanity today: the health and well-being of populations and the state of the natural environment. Mental Health disorders are on the rise across the world. A report commissioned by Lancet in 2018 estimated that 1.1 billion people are currently affected by adverse mental health issues (Chandra and Chand, 2018; Frankish et al., 2018). At the same time, the planet is being pushed to its limits from the effects of climate change and there is an ongoing biological annihilation (Ceballos et al., 2017). The implications of these issues are not only financial; they threaten the future of human civilization itself (Ceballos et al., 2017) as it depends upon the Earth's natural systems (Whitmee et al., 2015). It is now vital that governments, policy makers and practitioners across all sectors focus efforts on improving the human-nature relationship. Recognition of the importance of finding ways to improve the human relationship with the rest of nature for the well-being of people and the wider natural world is now international and reflected in responses to the United Nations Sustainable Development Goals (goal 3) (Chandra and Chand, 2018; Peacock and Brymer, 2019; Parsons et al., 2019; Sharma-Brymer and Brymer, 2019), “One Health” models of human, environmental and wildlife health (Rabinowitz et al., 2018) and clinical ecology (Nelson et al., 2019).

Some argue that globalization, the rise in technology, population growth, and the perceived diminution of nature's worth for human psychological, emotional and physical health has caused a disconnect between humanity and the rest of nature. As this disconnect continues and potentially grows, the prospects of achieving human well-being within the dominant economic development paradigm weakens. Vital alternative, sustainable, and integrated development paradigms are being developed that aim to re-address the balance between the human system and the Earth system (Rockström, 2015). Fortunately, research in this area continues to grow and we know a great deal more about the human-nature relationship, its benefits and ways to improve it (e.g., Lumber et al., 2017) than we did just a few years ago. The articles in this special edition clearly demonstrate this and provide hope that we will find a better way to relate to the rest of the natural world and consequently to ourselves.

It is now clear that the responsibility for mapping out the future for human health is not merely an issue for medicine and allied health. Perhaps more than any other issue affecting humanity, the future for the health of people and planet depends on multiple disciplines working together. This special edition reflects this notion with perspectives and evidence drawn from psychology, sport science, public health, environmental studies, biology, social science, forestry, education, occupational health, information technology, built environments, pharmaceutical and medical sciences, zoology, tourism, and philosophy. Researchers herald from the UK, Australia, United States, Finland, Norway, France, and Austria providing a wide, inclusive and multidisciplinary insight to this research area.

All of the papers argue that the human-nature relationship is an important one, one to understand, enhance, and protect. Human health and well-being benefits range from those that enhance flourishing and thriving to those where nature interactions protect against the onset of illness, to those where nature is an effective intervention for ill health. The contexts explored in this special edition are equally diverse and include the workplace (Hyvönen et al.), semi-natural or urban green spaces (Pasanen et al.; Wood et al.; Tracey et al.; Roe et al.) as well as wilder contexts (Niedermeier et al.), which all found nature experiences in these contexts beneficial to improving well-being. Importantly, Barnes et al.; Roe et al.; Schebella et al. and Wood et al. provide wider evidence of the link between the natural environment, biodiversity and well-being, and Hyvönen et al. show that nature should be included in models of workplace well-being. Additionally, there is recognition of the challenges of accessing nature and research on the use of nature-based guided imagery (Nguyen and Brymer) and simulations of natural scenes (Wooller et al.; Calogiuri et al.) find they are effective anxiety and stress management interventions. Roe et al. highlight well, highlight well, however, the need to understand the complexities of stress-management arguing that age and other demographic variables are important to consider.

The special issue supports and notes (e.g., Barnes et al.) the growing evidence that nature is good for well-being. The issue presents specific interventions (e.g., Nguyen and Brymer) and nature as a therapeutic environment (Tracey et al.). However, Barnes et al. and Summers and Vivian show how nature is still an unrecognized health resource despite evidence of the benefits from numerous sources, including large scale national campaigns such as 30 Days Wild which benefits well-being by improving nature connectedness (Richardson and McEwan).

We also see different concepts, theories and therapies considered in attempts to better understand and work with nature. For example, Schweitzer et al. argue that phenomenological and psychoanalytic perspectives offer a richness to understanding experiences, finding that nature is an integral part of the sense of self among people who considered nature as essential to their health and well-being. Stoic and Buddhist traditions are considered by Fabjanski and Brymer who argue mindful attention to natural patterns and rhythms, cognitive interventions and deconstructing and relinquishing anthropomorphic perceptions are key aspects to how nature enhances health and well-being. Acceptance and Commitment Therapy (ACT) was also combined with Adventure Therapy (AT) to explore ways of promoting the well-being of children “at-risk” (Tracey et al.). A review of Ecotherapy (Summers and Vivian) highlights the role of human-ecosystems interaction as a therapeutic device claiming that nature provides a service that is undervalued in ecological literature.

Evidence in this special edition, alongside an increasingly vast array of published work, seems to support a push for a health service built around the integration of human experience with nature (Natural England, 2009), and the need to improve and diversify nature-based provision for social prescribing to suit different contexts, preferences, resources and needs. Caution is rightly encouraged though by van Heezik and Brymer who question the prevalent use of nature as a commodity and reveal the often brushed aside tensions between human-nature relationship work and the need to protect the very thing that keeps us healthy. Often such challenging topics are avoided. Wood et al. and Schebella et al., demonstrate further why such questioning is so essential when they showed that biodiversity underpinned people's choice of favorite places and their perceptions of restorative impacts. Challenges also still exist in understanding mechanisms underpinning the well-being benefits of human-nature relationship (though some research is edging closer e.g., Richardson and McEwan; Lawton et al.) which reflect the need to move beyond the limitations exposed when examining traditional and well-established theories, such as Attention Restoration Theory and Biophilia.

Crucially, we need to understand more about how we can both enhance well-being through nature exposure and experiences, and become stewards of nature, working toward protecting it more effectively, and allowing nature to also flourish—developing a closer, connected relationship with the rest of the natural world. Continuing research in this area in an interdisciplinary and trans-disciplinary way is therefore vital. All too often researchers work within the safety of their own disciplines. Pioneers within this specialism should demonstrate (more often) how to work together across disciplines and showcase the fruits of their work widely and in ways that can be applied.

Despite the breadth of evidence, nature based solutions remain inexplicably absent from the dominant models of health, health behavior change (e.g., Gritti, 2017) and workplace well-being (Richardson et al., 2017). Yet this special issue presents clear evidence of the benefits of human embeddedness within the natural world (e.g., papers by Fabjanski and Brymer and Schweitzer et al.) and the importance of moving forward with a multidisciplinary approach. Both these perspectives (embeddedness and multidisciplinary work) can be seen to underpin the benefits, for example, of nature-based exercise (Wooller et al.) and engagement with nature's beauty (Richardson and McEwan). Research in this special edition demonstrates that the human-nature relationship as it pertains to health and well-being is clearly more nuanced than traditionally understood. How this relationship provides for such a broad impact on psychological health, including increased flourishing and decreases in a broad array of mental illness, needs further exploration. However, what seems clear is that much depends on understanding the relationship between activity, individual characteristics and environmental characteristics.

Future research should focus on two areas. Firstly, there is no human well-being without nature's well-being, and the threats to biodiversity, wildlife and the living planet are present and severe. In order to maximize the opportunities for both humans and nature to thrive, further research is needed to understand *how* the human-nature relationship works and following on from this, how best to improve the human-nature relationship. This will require investigations that recognize and explore the complexities of the human-nature relationship, acknowledge the role of meaning and meaning-making (Freeman et al., 2016; Freeman and Akhurst, 2018) and respond to this call for further research in a nuanced manner, avoiding reductionist or narrow tendencies. The continuation of interdisciplinary collaboration is therefore vital. Future research that provides a deeper understanding of the human-nature relationship has the potential to aid the development and improvement of these broader efforts. The continuation of current funding that supports these research needs and the expansion of funding opportunities in this area are therefore needed if current crises in health, mental health and our planetary future are to be addressed.

Secondly, there is an urgent need to find ways to improve the human-nature relationship through interventions, campaigns (Richardson and McEwan), activities, curricula, green infrastructure and urban design. Bringing together artists, planners, designers, and researchers to create places that afford a connection to nature. Such research should go beyond understanding to application, creating accessible and effective tools for practitioners from all aspects of human-environment interaction to address the human-nature relationship. An exemplar and catalyst for this movement is provided in the recommendations below.

## Recommendations

There will always be a need for further research and understanding, but owing to the crises in well-being and biodiversity a new relationship with nature, where nature and well-being are central determinants of human development, is needed now. Therefore, the research in the special issue can be distilled into a number of recommendations that recognize the importance of human-nature relationships for both human and nature's well-being:

- Everyday experiences of nature matter. Provide green spaces, close to home and work, with opportunities and prompts for people across the lifespan to notice nature and its beauty. See Richardson and McEwan and Roe et al.
- Encourage a broader range of seasonal experiences in nature, of various durations, at various times and calling on insight from a range of approaches to human-nature relationships (e.g., Stoic and Buddhist Traditions; nature connectedness). See Barnes et al.; Fabjanski and Brymer; and Richardson and McEwan.
- Provide habitats for a variety of wildlife. Biodiversity matters for human health. Micro-variables such as birds, plants, wildlife, and native species create a bond between people and natural places. See van Heezik and Brymer and Schebella et al.
- Activity in natural environments is good and better than in other environments. Provide opportunities to encourage walking and exercise in nature in residential and work contexts. Compared to indoor exercise there are additive benefits of a closer relationship with nature and reduced anxiety. See Lawton et al.; Wooller et al.; Hyvönen et al.; and Niedermeier et al.
- Provide nature based therapeutic environments. See Tracey et al. and Summers and Vivian
- For those with limited access to nature, provide imagery and VR alternatives. See Nguyen and Brymer and Calogiuri et al.

Together the articles in this special issue provide one bounded example of how interdisciplinary approaches to appreciating the nuances involved in uniting human and planetary health can help rethink the human-nature relationship and inform the international need for a perspective that positively impacts on the well-being of human beings and our planet. The evidence is clear; the well-being of future populations and the planet depends on a cross sector commitment and an authentic desire to refocus political and practical efforts on effective human-nature relationships.

## Author Contributions

All authors listed have made a substantial, direct and intellectual contribution to the work, and approved it for publication.

### Conflict of Interest Statement

The authors declare that the research was conducted in the absence of any commercial or financial relationships that could be construed as a potential conflict of interest.
